# Low, borderline and normal ankle-brachial index as a predictor of incidents outcomes in the Mediterranean based-population ARTPER cohort after 9 years follow-up

**DOI:** 10.1371/journal.pone.0209163

**Published:** 2019-01-23

**Authors:** M. Teresa Alzamora, Rosa Forés, Guillem Pera, José Miguel Baena-Díez, Marta Valverde, Pere Torán

**Affiliations:** 1 Centre d’Atenció Primària Riu Nord- Riu Sud Santa Coloma de Gramenet, Direcció d’Atenció Primària Barcelonés Nord i Maresme, Institut Català de la Salut, Barcelona, Spain; 2 Unitat de Suport a la Recerca Metropolitana Nord, Institut Universitari d’Investigació en Atenció Primària Jordi Gol (IDIAP Jordi Gol), Mataró, Spain; 3 Universitat Autònoma de Barcelona, Bellaterra (Cerdanyola del Vallès), Spain; 4 Centre d’Atenció Primària La Marina, Direcció d’Atenció Primària Barcelona Ciutat, Institut Català de la Salut, Barcelona, Spain; 5 Unitat de Suport a la Recerca Barcelona, Institut Universitari d’Investigació en Atenció Primària Jordi Gol (IDIAP Jordi Gol), Barcelona, Spain; 6 Hospital Nostra Senyora de Meritxell, Escaldes-Engordany, Andorra; Medical University Innsbruck, AUSTRIA

## Abstract

**Background:**

Guidelines recommended adopting the same cardiovascular risk modification strategies used for coronary disease in case of low Ankle-brachial index (ABI), but here exist few studies on long-term cardiovascular outcomes in patients with borderline ABI and even fewer on the general population.

**Aim:**

The aim of the present study was to analyze the relationship between long-term cardiovascular events and low, borderline and normal ABI after a 9-year follow up of a Mediterranean population with low cardiovascular risk.

**Design and setting:**

A population-based prospective cohort study was performed in the province of Barcelona, Spain.

**Method:**

A total of 3,786 subjects >49 years were recruited from 2006–2008. Baseline ABI was 1.08 ± 0.16. Subjects were followed from the time of enrollment to the end of follow-up in 2016 via phone calls every 6 months, systematic reviews of primary-care and hospital medical records and analysis of the SIDIAP (Information System for Primary Care Research) database to confirm the possible appearance of cardiovascular events.

**Results:**

3146 individuals participated in the study. 2,420 (77%) subjects had normal ABI, 524 (17%) had borderline ABI, and 202 (6.4%) had low ABI.

In comparison with normal and borderline subjects, patients with lower ABI had more comorbidities, such as hypertension, hypercholesterolemia and diabetes.

Cumulative MACE incidence at 9 years was 20% in patients with low ABI, 6% in borderline ABI and 5% in normal ABI.

The annual MACE incidence after 9 years follow-up was significantly higher in people with low ABI (26.9/1000py) (p<0.001) than in borderline (6.6/1000py) and in normal ABI (5.6/1000py).

Subjects with borderline ABI are at significantly higher risk for coronary disease (HR: 1.58; 95% CI: 1.02–2, 43; p = 0,040) compared to subjects with normal ABI, after adjustment.

**Conclusion:**

The results of the present study support that low ABI was independently associated with higher incidence of MACE, ICE, cardiovascular and no cardiovascular mortality; while borderline ABI had significantly moderate risk for coronary disease than normal ABI.

## Introduction

The Ankle-Brachial Index (ABI) is a simple and non-invasive tool used to diagnose Peripheral arterial disease (PAD). An ABI of <0.9 has been considered abnormal and has been not only associated with the diagnosis of PAD, but has been also a marker of incident cardiovascular events and mortality in both symptomatic and asymptomatic forms of PAD [1–7]. It has been repeatedly associated with a three to six times greater risk of cardiovascular events and mortality [8].

On the other hand, to add ABI <0.9 to the cardiovascular risk scales improve the predictive capacity of them, as a tool to help reclassify coronary risk. This increased risk is independent from traditional cardiovascular risk factors [8, 9].

It is very important to determine the epidemiology of this disease since PAD prevalence is increasing globally in high-, middle- and low-income countries [10]. Between 8% and 23% of people over 50 years of age are affected by PAD [10–11]. Management of PAD includes intervention targeted at specific arterial symptoms as well as general prevention of cardiovascular risk.

Several studies carried out in countries with high and low rates of cardiovascular disease have detected a high incidence of cardiovascular events and mortality in patients with PAD [1–7].

The evidence is strong enough that guidelines recommend adopting the same cardiovascular risk modification strategies used for coronary arterial disease in cases of PAD [12–15].

PAD, however, is underdiagnosed (in spite of significant efforts made by primary health care centers) [12]. This may be attributed to the fact that up to two-thirds of patients with PAD in the community are asymptomatic [13].

The American College of Cardiology and the American Heart Association (ACC/AHA) guidelines on management of PAD patients recommend that an ankle-brachial index between 0.90 and 0.99 be considered borderline; patients with borderline ABI should be considered a high-risk group, as should patients with abnormal ABI (<0.9) [14,15]. Patients with borderline ABI (0.90–.99) should undergo further diagnostic tests.

However, there exist few studies on long-term future cardiovascular events in patients with borderline ABI [16, 17] and even fewer on the general population.

To resolve this issue, we evaluated the relationship between future cardiovascular events and low, borderline and normal ABI in a Mediterranean population-based cohort (ARTPER).

## Material and methods

ARTPER study was approved by the local Ethics Committee (IDIAP Jordi Gol Foundation of Investigation in Primary Care and Instituto de Salud Carlos III) Committee in July 2006 and the approval was renewed in September 2011 and March 2016. All the patients provided informed written consent.

### Study population

**Fig 1:**
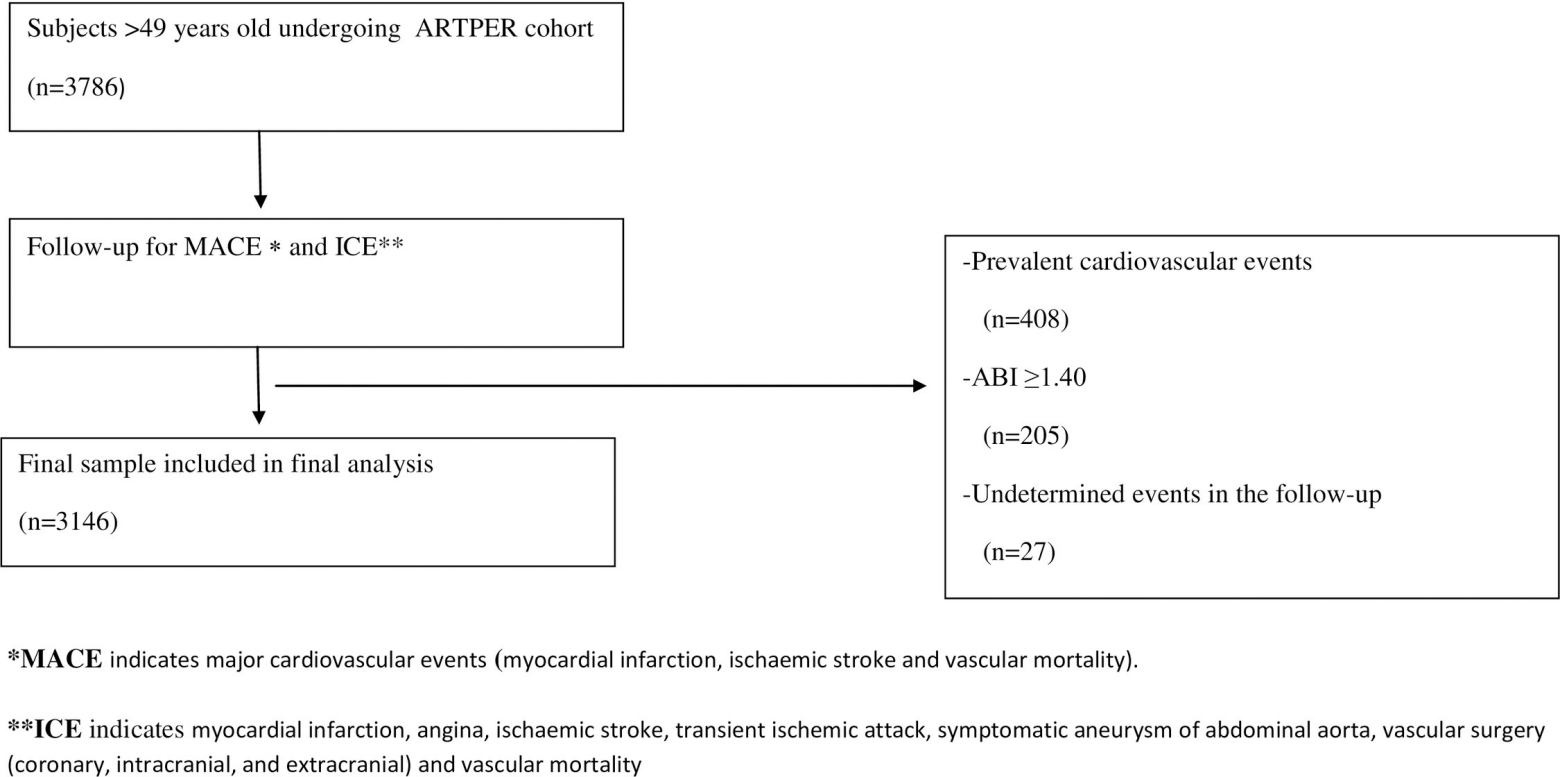
Flow-chart of study participants.

The design of the ARTPER study has previously been described and is summarized in the patient flow-chart **Fig 1.** [18]. The study is an ongoing prospective observational cohort initiated in October 2006.

Baseline ABI was measured in 3,786 randomly selected patients older than 49 years of age who were ascribed to 28 primary health care centers in the Barcelona area from September 2006 through June 2008.

### Diagnosis and definition

ABI was calculated to estimate lower-extremity arterial disease. Supine systolic blood pressures were measured in both arms. Ankle systolic blood pressures in bilateral posterior tibial and dorsalis pedis arteries were obtained using an aneroid sphygmomanometer and a standard Doppler ultrasound device with an 8 MHz probe. Leg-specific ABI was calculated by dividing the higher of the two systolic pressure readings taken from the legs by the higher of the two systolic pressure readings taken from the arms.

PAD was defined as ABI <0.9, borderline ABI 0.90–0.99, normal ABI 1.00–1.39 and arterial calcification ≥1.40.

Incident vascular events (ICE) were defined as a myocardial infarction, angina, stroke, transient ischemic attack, symptomatic aneurysm of abdominal aorta, vascular surgery (coronary, intracranial, and extracranial) and mortality. Deaths were adjusted for the presence or absence of a cardiovascular cause.

Incident major adverse cardiovascular events (MACE) included myocardial infarction, stroke and vascular mortality.

### Follow-up and endpoint adjudication

Subjects were followed from the time of enrollment to the end of follow-up in November 2016 via phone calls every 6 months, systematic reviews of primary-care and hospital medical records and analysis of the SIDIAP (Information System for Primary Care Research) database.

A medical committee comprising members who carry out routine clinical practice reviewed all clinical incident events, which were grouped as follows: coronary disease (myocardial infarction or angina), cerebrovascular disease (stroke or transient ischemic attack), symptomatic aneurysm of the abdominal aorta (SAAA), vascular surgery, cardiovascular morbidity (any of the previous four types), vascular mortality (presence of vascular cause), non-vascular mortality (absence of vascular cause), overall mortality (vascular or non-vascular), or morbimortality (any of the events). Only the first episode for each type of event was taken into account even though any recurring events were recorded. Any patient that had had an event at the time of or prior to recruitment was excluded from the analysis.

## Statistical analysis

All ABI group comparisons have been made in 2 pairs: normal ABI vs. low ABI and normal ABI vs. borderline ABI. For categorical variables frequencies and percentage are shown and for continuous variables means and standard deviations, being tested using chi squared and Student’t-test respectively. MACE and ICE Nelson-Aalen cumulative hazard functions have been estimated and plotted, including their 95% confidence intervals, for each of the 3 ABI groups. The percentage of subjects in each ABI group that at the end of follow-up had MACE (or ICE) has been computed dividing the new MACE (or ICE) diagnoses by the baseline number of subjects in each group. At the end of follow-up, incidence for MACE (or ICE) has been computed using only the first episode of MACE (or ICE), with the time to the first episode or end of follow-up (if not a case) from the recruitment as the time being at risk for each subject. Log-rank tests have been used to compare the survivor functions between the different ABI groups. For different cardiovascular events, different proportional risk Cox regression models have been used to estimate the hazard ratio (HR) of having these events depending on the ABI group, raw and adjusted by age, gender, smoking, obesity, hypertension, diabetes and hypercholesterolemia. Incidence of PAD among each ABI group was computed after excluding those with baseline PAD. HRs have been computed to assess the risk of having borderline PAD vs. normal PAD on developing PAD, raw and adjusted by age, gender, smoking, obesity, hypertension, diabetes and hypercholesterolemia. All the tests performed were bilateral and the significance was < 0.05. We used the Stata v15 to perform the statistical analysis.

## Results

### Baseline characteristics by ankle-brachial index (ABI)

The study population included 3,786 subjects with a mean age of 65 ± 9 years and 54% (n = 2040) were female. Average ABI for the entire population was 1.08 ± 0.16.

Of the 3786 subjects, 640 were excluded in the analyzed for this study (408 for prevalent cardiovascular events, 205 for arterial calcification (ABI ≥ 1.4) and 27 for undetermined events in the follow-up) **Fig 1**, leaving a total of 3,146 subjects with a mean age of 64 ± 9 and 57% were women (n = 1807).

Subjects were followed up during an average of 8.7 years (SD 1.8, range 51 days-10.3 years), adding up to 27,469 person-years (py).

Of 3,146 patients, a total of 2,420 (77%) subjects had normal ABI, 524 (17%) patients had borderline ABI, and 202 (6.4%) patients had low ABI.

Mean ABI was 1.11 ± 0.09; 0.95 ± 0.03 and 0.74 ± 0.13 for normal, borderline and low ABI, respectively.

In comparison with normal and borderline subjects, patients with lower ABI had more comorbidities, such as hypertension, hypercholesterolemia and diabetes.

There were no differences between normal and borderline ABI groups except in age, total cholesterol and HDL cholesterol. **Table 1**.

**Table 1:** Baseline characteristics of the patients according to ABI classification.

	Normal ABI (1.00–1.39)		Borderline ABI (0.90–0.99)		Low ABI (<0.90)		p values	
	n =	2420	n =	524	n =	202	Normal vs Borderline	Normal vs Low
Age (years)	64	±8	65	±9	70	±10	0,015	<0.001
Women, n (%)	1396	(58%)	320	(61%)	91	(45%)	0,154	<0.001
General obesity *, n (%)								
Men	319	(31%)	72	(35%)	32	(29%)	0,250	0,610
Women	545	(39%)	134	(42%)	43	(47%)	0,364	0,125
Abdominal obesity **, n (%)								
Men	461	(45%)	90	(44%)	54	(49%)	0,742	0,511
Women	984	(71%)	238	(75%)	72	(79%)	0,150	0,098
Ever smoker, n (%)	985	(41%)	232	(44%)	116	(57%)	0,132	<0.001
ABI value	1,12	±0,08	0,95	±0,03	0,76	±0,13	<0.001	<0.001
Medical records, n (%)								
Hypertension	1000	(41%)	232	(44%)	127	(63%)	0,214	<0.001
Hypercholesterolemia	1068	(44%)	232	(44%)	112	(55%)	0,952	0,002
Diabetes	326	(13%)	63	(12%)	61	(30%)	0,375	<0.001
Blood analysis								
Total cholesterol (mg/dl)	218	±38	223	±37	215	±43	0,010	0,285
HDL cholesterol (mg/dl)	56	±14	58	±15	54	±15	0,028	0,009
LDL cholesterol (mg/dl)	137	±33	140	±33	133	±36	0,111	0,102
Treatments (medical records), n (%)								
Antihypertensives	876	(36%)	206	(39%)	115	(57%)	0,180	<0.001
Antiplatelet/anticoagulant	192	(8%)	46	(9%)	53	(26%)	0,520	<0.001
Hypolipidemics	618	(26%)	142	(27%)	75	(37%)	0,459	<0.001
Hypoglycemics	251	(10%)	41	(8%)	55	(27%)	0,077	<0.001
Cardiovascular risk								
REGICOR***	5,6	±3,5	5,9	±3,9	8,3	±5,3	0,100	<0.001
SCORE***	3,0	±3,1	3,3	±3,6	5,1	±5,5	0,115	<0.001

* Defined as body mass index≥30 Kg/m^2^

** Defined as

*** REGICOR and SCORE only computed among those younger than 75 and 66 years respectively.

HDL, high density lipoprotein. LDL, low density lipoprotein.

Missing values: general obesity (4), abdominal obesity (22), total cholesterol (25), LDL cholesterol (30).

Results are mean ± standard deviation, unless otherwise stated.

### All-cause MACE based on ABI category

**Fig 2:**
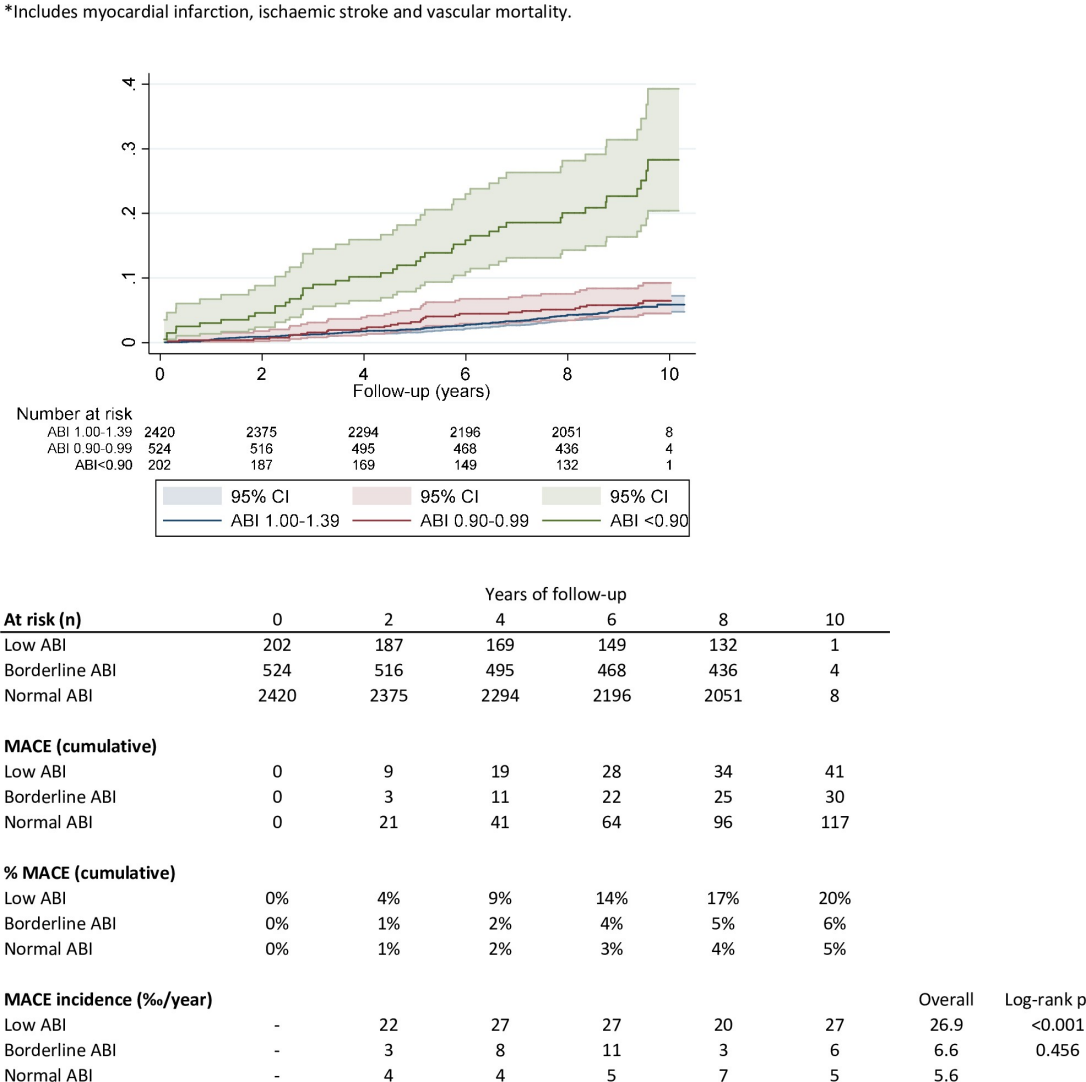
Cumulative hazard of MACE for lower, borderline and normal ABI.

During a mean follow-up period of 8.7 years, there were 188 MACEs (80 myocardial infarction, 71 strokes and 73 vascular mortalities): 41 in patients with low ABI, 30 in patients with borderline ABI and 117 in patients with normal ABI. **Fig 2.**

This represents a cumulative MACE incidence at 10 years of 20% in patients with low ABI, 6% in borderline ABI and 5% in normal ABI.

The annual MACE incidence after 10 years follow-up was significantly higher in people with low ABI (26.9/1000py) (p<0.001) than in borderline (6.6/1000py) and in normal ABI (5.6/1000py). Differences between borderline and normal ABI were not statistical significant (p = 0.456).

### All-cause ICE based on ABI category

**Fig 3:**
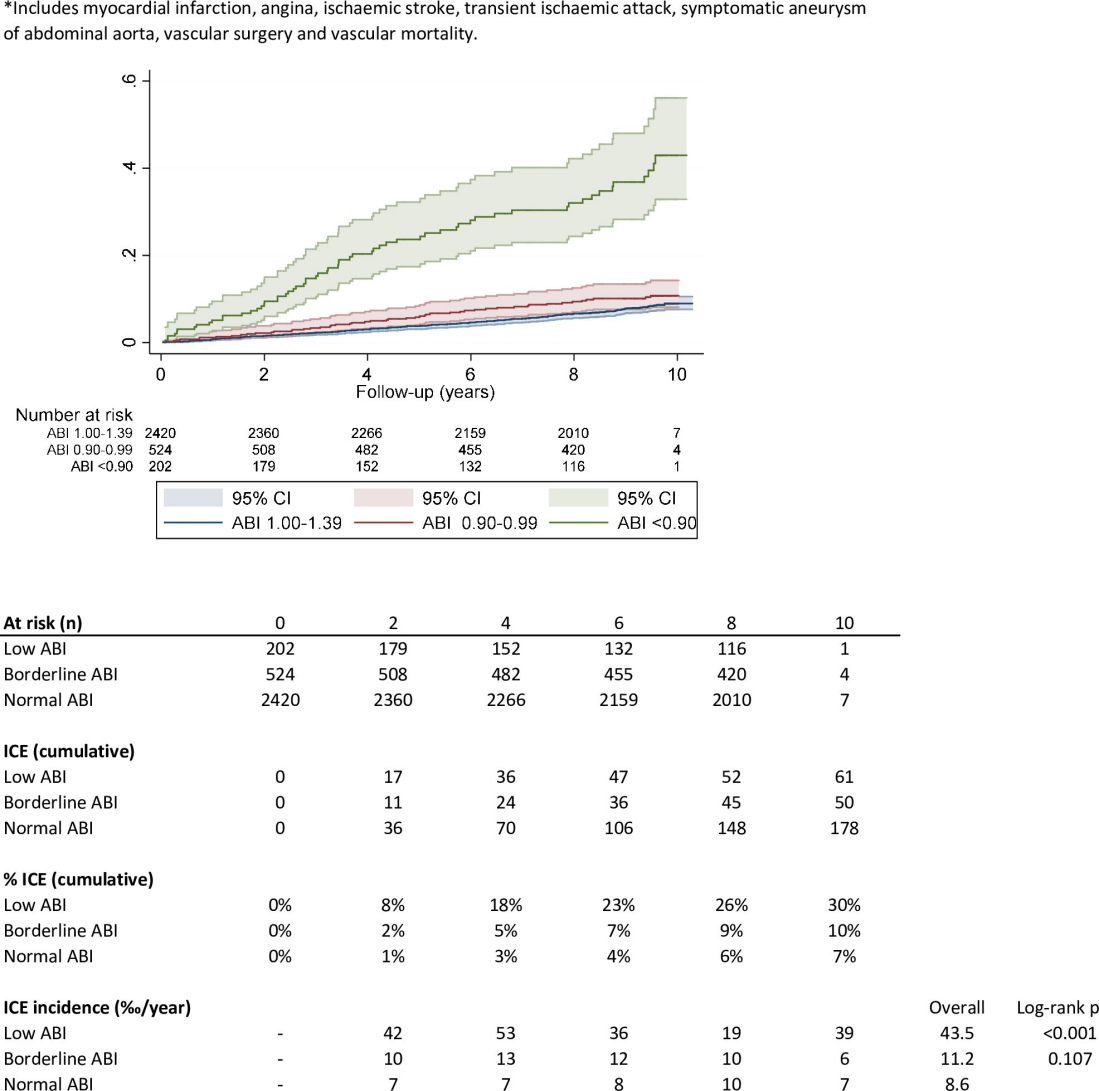
Cumulative hazard of ICE for lower, borderline and normal ABI.

In the same period of follow-up, there were 289 ICEs, 61 in patients with low ABI, 50 in patients with borderline ABI and 178 in patients with normal ABI. **Fig 3.**

This represents a cumulative ICE incidence at 10 years of 30% in patients with low ABI, 10% in borderline ABI and 7% in normal ABI.

The annual ICE incidence after 10 years follow-up was significantly higher in people with low ABI (43.5/1000py) (p<0.001) than in borderline (11.2/1000py) and in normal ABI (8.6/1000py). Differences between borderline and normal ABI were not statistical significant (p = 0.107)

### All-cause morbimortality

In multivariate Cox proportional regression analysis, we evaluated ABI as a predictor for the clinical outcomes MACE and ICE.

Subjects with low ABI are at significantly higher risk for all event types except for cerebrovascular disease and non-vascular mortality compared to patients with normal ABI. PAD increased the risk of MACE more than two fold (HR = 2.43, 95% CI 1.67–3.56; p<0.01), as well as the risk of coronary disease (HR = 2.99, 95% CI 1.91–4.60; p<0.01) and vascular mortality (HR = 3.13, 95% CI 1.79–5.48; p<0.01). **Table 2**.

**Table 2:** Cox proportional regression analysis for clinical outcomes.

	Normal vs Borderline			Normal vs Low				
	HR	95%CI		p value	HR	95%CI		p value
MACE raw	1,16	0,78	1,74	0,456	4,88	3,42	6,96	<0.001
MACE adjusted*	**1,04**	0,69	1,55	0,866	**2,43**	1,67	3,56	<0.001
Myocardial infarction raw	1,39	0,77	2,54	0,276	5,91	3,49	9,99	<0.001
Myocardial infarction adjusted	1,32	0,72	2,42	0,363	3,20	1,82	5,64	<0.001
Coronary disease** raw	1,60	1,04	2,45	0,033	5,13	3,37	7,80	<0.001
Coronary disease** adjusted	**1,58**	1,02	2,43	0,040	**2,99**	1,91	4,69	<0.001
Stroke raw	0,78	0,38	1,59	0,494	2,61	1,33	5,14	0,005
Stroke adjusted	0,72	0,35	1,47	0,364	1,31	0,65	2,66	0,455
Cerebrovascular disease*** raw	0,77	0,40	1,45	0,417	3,16	1,80	5,54	<0.001
Cerebrovascular disease*** adjusted	**0,69**	0,36	1,31	0,252	**1,55**	0,86	2,79	0,149
Morbidity raw	1,22	0,85	1,75	0,290	4,19	2,96	5,94	<0.001
Morbidity adjusted	1,15	0,80	1,66	0,447	2,29	1,58	3,31	<0.001
Vascular intervention raw	1,45	0,83	2,54	0,197	6,24	3,81	10,21	<0.001
Vascular intervention adjusted	1,49	0,85	2,62	0,168	4,06	2,38	6,91	<0.001
Vascular mortality raw	1,59	0,85	3,00	0,148	8,22	4,88	13,84	<0.001
Vascular mortality adjusted	1,25	0,65	2,38	0,500	3,13	1,79	5,48	<0.001
Non-vascular mortality raw	0,96	0,66	1,39	0,834	2,88	2,00	4,15	<0.001
Non-vascular mortality adjusted	0,82	0,56	1,19	0,300	1,32	0,90	1,94	0,156
Morbimortality raw	1,11	0,87	1,41	0,397	4,18	3,33	5,24	<0.001
Morbimortality adjusted	1,01	0,79	1,28	0,960	2,12	1,67	2,70	<0.001

* Adjusted by age, gender, smoking, obesity, hypertension, diabetes and hypercholesterolemia.

** Includes myocardialinfarction and angina

*** Incudes stroke and transient ischaemic attack

Subjects with borderline ABI are at significantly higher risk for coronary disease (HR: 1.58; 95% CI: 1.02–2, 43; p = 0,040) compared to subjects with normal ABI, after adjustment.

### PAD incidence

PAD incidence after 5 years of follow-up population-based cohort ARTPER was previously calculated [19]. We considered PAD incident when the second cross section Ankle brachial Index was <0.9 in any of the lower limbs, with normal baseline (0.9 to 1.4).

The incidence of PAD, at 5 years follow-up was remarkably higher in subjects with borderline ABI (n = 51; 12.3%) than in those with normal ABI (n = 44; 2.2%) (p<0.001). PAD was (HR = 5.44, 95% CI 3.63–8.15; p = 0.000). After adjustment by (age, gender, smoking, obesity, hypertension, diabetes and hypercholesterolemia), subjects with borderline ABI had significant higher risk for PAD compared with normal ABI subjects (HR = 4.21, 95% CI 2.76–6.40; p = 0.000).

## Discussion

Up to the present time, the ARTPER low cardiovascular risk population cohort evaluated the incidence of vascular events as well as the improvement in the reclassification of the cardiovascular risk scales to the addition of the ABI <0.9 in them [5, 9].

The present study was performed to evaluate incident events in low, borderline and normal ABI subjects.

Our main findings were as follows: (1) general population subjects with low ABI presented the most incident events of all-cause morbimortality even after adjusting for classical cardiovascular risk factors (with the exception of stroke); (2) A total of 16.7% of subjects with normal ABI were reclassified as having borderline ABI (n = 524).

(3) General population subjects with borderline ABI had significantly higher incident coronary disease results, even after adjustment. (4) Borderline ABI was independently associated with PAD incidence in the five-year follow-up.

While low ABI has been examined in previous population-based studies [10, 11, 15, 18], the prevalence of borderline ABI has not been investigated as extensively. In our study, the prevalence of low and borderline ABI in patients >49 years old was 6.4% and 16.6%, respectively. Our results are lower than those found in patients from countries with high rates of cardiovascular disease, such as the United States (8.7% and 27.8%, respectively) [20].

In previous studies, low ABI has been associated with an increased risk of incident outcome and all-cause mortality in selected and general populations [1, 2, 3, 4, 5, 6, 10].

In a Cardiovascular health study (5,714 participants) patients with ABI <0.9 revealed an increased all-cause mortality of 1.62 and an increased cardiovascular mortality of 2.03 [21].

A multi-ethnic study of atherosclerosis (6,570 participants) identified an increased prevalence of subclinical atherosclerosis among women and men with borderline ABI [22].

In a previous analysis of the low cardiovascular risk ARTPER cohort [5], the importance of low ABI as an independent risk factor was confirmed. Low ABI had a two times greater risk of coronary disease (HR  =  2.0) and an increased risk of vascular surgery (HR  =  5.6) and mortality (HR  =  1.8).

On the other hand, the prognostic significance of borderline ABI has not been investigated as extensively in population-based studies.

The ankle-brachial index collaboration meta-analysis (which included more than 50,000 subjects from 16 international studies) [8] already found that subjects with an ABI between 0.91 and 1.10 were at a slightly increased risk. Based on these findings, the American Heart Association modified the definition of normal ABI values and created the definition of borderline ABI [23]. Nevertheless, this prognostic has not been fully clarified for the general population. In a Japanese retrospective hospital-based cohort study, Tanaka et al. found that borderline ABI was independently and statistically significant associated with a high risk of all-cause mortality (HR 2.27) and cardiovascular events (HR 1.38;) [16].

Natsuaky et al. also found that borderline ABI was associated with high risk of mortality in diabetic patients (3,981) [17].

In the present study performed in a Mediterranean based-population cohort with a low cardiovascular risk, we found that subjects with borderline ABI are at significantly greater risk for coronary disease (HR: 1.58) but we did not find differences for others events. Our data is similar to that yielded by the long-term general Japanese population-based cohort (2,954) (Hisayana Study) that found no relationship between borderline ABI and cardiovascular events [24].

One explanation of the lack of associations between borderline ABI and ICE or MACE could be that both are small population-based cohorts and both studies excluded subjects with previous cardiovascular events. Some authors studied selected populations, such as diabetics or a hospital-population including ischaemic heart disease, and did not exclude subjects with prevalent events [16, 17].

As some authors suggest, perhaps the optimal cutoff point for the diagnosis of borderline ABI should be narrowed to: ABI 0.91–0.94 and 0.95–0.99 (n = 238 vs. n = 286 respectively) [16]. We don’t analyze the optimal cutoff point because this represented a small size in our cohort.

The present study has several strong points. Firstly, this is an ongoing prospective observational-based cohort population study with a mean follow-up period of 8.7 years. Secondly, all clinical incident events have been checked by a medical committee comprising members who perform routine clinical practice. Thirdly, any patient that had had an event at the time of or prior to recruitment was excluded from the analysis.

Concerning to the limitations of the study, first, we did not know the real data of beginning cardiovascular risk factors as diabetes, hypertension and hypercholesterolemia and these risk factors might affect the incident cardiovascular events. Second, we did not enroll subjects who were younger than 49 years old and consequently ours results cannot be applied in young people. Third, relatively small sample size of patients with low and borderline ABI in addition to the limited number of events. This could lead to a lack of power to detect differences between, mainly, borderline vs normal subjects, and even more for the adjusted models. However, first: the HRs of the normal vs borderline comparisons are always lower than those corresponding to the normal vs low comparisons, second: the differences in the HRs using raw or adjusted models are little for the normal vs borderline comparisons, and third: although HRs decrease when adjusting the normal vs low comparisons, most of them are still statistical significant (p<0.05).

Patients with elevated ABI were excluded as in other studies (17,20 because their clinical significance and treatment are different compared to patient with low ABI and we did not perform toe-brachial index to know really if patients with ABI ≥1.4 have PAD or not.

## Conclusions

In conclusion, the results of the present study support that low ABI was independently associated with higher incidence of MACE, ICE, cardiovascular and no cardiovascular mortality; while borderline ABI had significantly moderate risk for coronary disease than normal ABI.

## Supporting information

S1 Fig CEIC 2006. (DOC)

S2 Fig CEIC 2011. (PDF)

S3 Fig CEIC 2016. (PDF)

S1 File Additional information from the study database. (XLS)
