# Supplementary material for: Low, borderline and normal ankle-brachial index as a predictor of incidents outcomes in the Mediterranean based-population ARTPER cohort after 9 years follow-up
Source: PLoS One. 2019 Jan 23;14(1):e0209163. doi: 10.1371/journal.pone.0209163 (PMC6343871; doi:10.1371/journal.pone.0209163)
Supplement: S1 Fig — (DOC) [file pone.0209163.s001.doc]

# INFORME DEL COMITÉ ÉTICO DE INVESTIGACIÓN CLÍNICA

Ester Amado Guirado, secretaria del Comité Ético de Investigación Clínica de la Fundación Jordi Gol i Gurina.

**CERTIFICA:**

Que este Comité en su reunión del día 28 de Junio de 2006, ha evaluado el proyecto de investigación **(P06/28)** titulado *“Prevalencia de la arteriopatia periférica y valor predictivo de sus formas silentes en relación a la morbi-mortalidad cardiovascular. Estudio multicénctrico poblacional”* presentado por la Dra. Maria Teresa Alzamora Sas.

Considerando que respeta los principios éticos y metodológicos para que pueda ser llevado a cabo, por lo que ha acordado dar su aprobación definitiva al proyecto anteriormente mencionado.

Lo que firmo en Barcelona a 4 de Julio de 2006.

p.o
