# Supplementary material for: Low, borderline and normal ankle-brachial index as a predictor of incidents outcomes in the Mediterranean based-population ARTPER cohort after 9 years follow-up
Source: PLoS One. 2019 Jan 23;14(1):e0209163. doi: 10.1371/journal.pone.0209163 (PMC6343871; doi:10.1371/journal.pone.0209163)
Supplement: S2 Fig — (PDF) [file pone.0209163.s002.pdf]

IDIAP
Jordi Gol

INFORME DEL COMITÈ ÈTIC D'INVESTIGACIO CLÍNICA

Gemma Rodríguez Palomar, secretaria del Comitè Étic d' Investigació Clínica del l'IDIAP Jordi Gol i Gurina.

CERTIFICA :

Que aquest Comitè en la reunió del mes de setembre de 2011, despres d'avaluar per petició de la Investigadora Principal Mª Teresa Alzamora el projecte d'investigació **(P11/61)** titulat: **Incidencia de arteriopatía periférica y relación con factores de riesgo cardiovascular y morbimortalidad cardiovascular a los 5 años de seguimiento de la cohorte poblacional ARTPER.**

Considera que respecta els requisits ètics de confidencialitat i de bona pràctica clinica vigents.

Barcelona setembre 2011.
