# Supplementary material for: Low, borderline and normal ankle-brachial index as a predictor of incidents outcomes in the Mediterranean based-population ARTPER cohort after 9 years follow-up
Source: PLoS One. 2019 Jan 23;14(1):e0209163. doi: 10.1371/journal.pone.0209163 (PMC6343871; doi:10.1371/journal.pone.0209163)
Supplement: S3 Fig — (PDF) [file pone.0209163.s003.pdf]

IDIAP Jordi Gol

INFORME DEL COMITÈ ÈTIC D'INVESTIGACIÓ CLÍNICA

Rosa Morros Pedrós, Presidenta del Comitè Ètic d'Investigació Clínica de l'IDIAP Jordi Gol.

CERTIFICA:

Que aquest Comitè en la reunió del dia 02/03/2016, ha avaluat el projecte ***Morbimortalidad cardiovascular y no cardiovascular a los 10 años de seguimiento de la cohorte poblacional ARTPER*** amb el codi **P16/014** presentat per l'investigador/a **Mª Teresa Alzamora Sas.**

Considera que respecta els requisits ètics de confidencialitat i de bona pràctica clínica vigents.

Barcelona, a 08/03/2016
